# Small pelagics in a changing ocean: biological responses of sardine early stages to warming

**DOI:** 10.1093/conphys/cow017

**Published:** 2016-05-17

**Authors:** Filipa Faleiro, Marta Pimentel, Maria Rita Pegado, Regina Bispo, Ana Rita Lopes, Mário S. Diniz, Rui Rosa

**Affiliations:** 1 MARE – Marine and Environmental Sciences Centre, Faculdade de Ciências, Universidade de Lisboa, Laboratório Marítimo da Guia, Av. Nossa Senhora do Cabo 939, Cascais 2750-374, Portugal; 2 Instituto Ciências Biomédicas Abel Salazar, Universidade do Porto, Largo Prof. Abel Salazar 2, Porto 4099-003, Portugal; 3 MARE – Marine and Environmental Sciences Centre, ISPA – Instituto Universitário, Rua Jardim do Tabaco 34, Lisboa 1149-041, Portugal; 4 UCIBIO, REQUIMTE, Departamento de Química, Faculdade de Ciências e Tecnologia, Universidade NOVA de Lisboa, Quinta da Torre, Caparica 2829-516, Portugal

**Keywords:** Heat shock response, lipid peroxidation, metabolism, ocean warming, *Sardina pilchardus*, thermal tolerance

## Abstract

Small pelagic fishes are known to respond rapidly to changes in ocean climate. In this study, we evaluate the effects of future environmental warming (+2°C) during the early ontogeny of the European sardine, *Sardina pilchardus*. Warming reduced the survival of 30-day-old larvae by half. Length at hatching increased with temperature as expected, but no significant effect was observed on the length and growth at 30 days post-hatching. Warming did not significantly affect the thermal tolerance of sardine larvae, even though the mean lethal temperature increased by 1°C. In the warm conditions, sardine larvae showed signs of thermal stress, indicated by a pronounced increase in larval metabolism (*Q*_10_ = 7.9) and a 45% increase in the heat shock response. Lipid peroxidation was not significantly affected by the higher temperature, even though the mean value doubled. Warming did not affect the time larvae spent swimming, but decreased by 36% the frequency of prey attacks. Given the key role of these small pelagics in the trophic dynamics off the Western Iberian upwelling ecosystem, the negative effects of warming on the early stages may have important implications for fish recruitment and ecosystem structure.

## Abstract (precis)

Under a future warming scenario, sardine (Sardina pilchardus) larvae showed signs of thermal stress, namely a reduced survival, a steep increase of metabolic rates and a rise in heat shock response.

## Introduction

The importance of small pelagic fishes in marine ecosystems goes far beyond what their size would suggest. In many of the highly productive ecosystems of the world, particularly in coastal upwelling regions, the food web is characterized by a ‘wasp-waist’ richness pattern. The lower and higher trophic levels contain a higher species richness than intermediate levels, which are dominated by only one or a few small pelagic fish species (Bakun, 1996). These small and planktotrophic fishes thus play a crucial role in the trophic dynamics of marine ecosystems, either by top-down controlling planktonic organisms or bottom-up controlling predators (Cury *et al.*, 2000). Besides their important ecological role, small pelagic fishes are also a valuable component of fisheries all over the world, representing over a third of the global yield of marine fishes and contributing significantly to the economy of many countries (Hunter and Alheit, 1995; Pikitch *et al.*, 2014).

Small pelagic fish populations respond rapidly to changes in ocean climate (Hunter and Alheit, 1995) and have experienced substantial changes in their distribution and abundance over decadal time scales (Lehodey *et al.*, 2006). For instance, populations in the North Atlantic are fluctuating in synchrony with sea temperature oscillations and shifting their distributional borders polewards (Alheit *et al.*, 2012). In the South-East Pacific, the first reaction of small pelagics to El Niño is to move southwards, away from the warm waters (Lehodey *et al.*, 2006). These shifts in distribution and abundance of small pelagics seem to be coupled strongly to changes in water temperature, but it is highly probable that they result from a mixture of climate-driven mechanisms, such as primary productivity and prey availability (Peck *et al.*, 2013).

At a time when Earth’s climate is warming at an unprecedented rate, with forecasts estimating a mean increase of 0.8–3.1°C in sea surface temperature by the end of the century (Collins *et al.*, 2013), it is essential to understand how ocean warming may further impact the distribution and abundance of small pelagic fishes and how this may affect the trophic relations and the structure of marine ecosystems. As the thermal limits of marine organisms are approached or even exceeded, biological processes, such as metabolism, growth, feeding, reproduction and behaviour, may be negatively affected, compromising the overall fitness and survival of the species (e.g. Roessig *et al.*, 2004; Pörtner *et al.*, 2005; Byrne, 2011). Early stages are expected to be the most vulnerable to ocean warming and environmental stress and may constitute a bottleneck for the persistence of species in a changing ocean. Warming has been shown to have a significant impact during the early ontogeny of several marine fishes, namely on survival, growth, development, metabolism, thermal tolerance and the incidence of skeletal deformities (e.g. Pankhurst and Munday, 2011; Munday *et al.*, 2012; Aurélio *et al.*, 2013; Pimentel *et al.*, 2014). Although some studies have projected the potential impact of future warming on the biogeography of some pelagic fish populations based on past field observations and modelling (e.g. Rijnsdorp *et al.*, 2009; Albouy *et al.*, 2013; Maynou *et al.*, 2014), the biological responses of the early stages of small pelagic fishes to future warming remain, to the best of our knowledge, unknown. The life-history characteristics of these fishes (including high mobility, early sexual maturity, high fecundity, year-round spawning if conditions are suitable, and short lifespan) make them highly sensitive to environmental forcing and, thus, an ideal subject for climate change studies (Hunter and Alheit, 1995).

The aim of this study was to evaluate the potential effects of ocean warming on the early stages of the European sardine, *Sardina pilchardus*, one of the most abundant small pelagic fishes in the world. In order to understand how sardine early stages may respond to future changes in ocean temperature, we evaluated the impact of warming (+2°C) on the survival, growth, metabolism, thermal tolerance, behaviour, heat shock response and lipid peroxidation in sardine larvae.

## Materials and methods

### Larval rearing

*Sardina pilchardus* eggs were obtained from adult fish captured off the western Iberian Peninsula between 2009 and 2011, and maintained since then at Oceanário de Lisboa (Lisboa, Portugal). Fish spawning was naturally induced by photothermal and dietary manipulation. Eggs spawned in February and March 2013 were used in this experiment. In the morning following each spawning, eggs were immediately transferred to Laboratório Marítimo da Guia (Cascais, Portugal) and randomly acclimated for 1 month to one of two different thermal scenarios: (i) the mean temperature during the peak of the spawning season (15°C); or (ii) the respective warming scenario (+2°C, 17°C). In Portuguese waters, sardine spawning occurs mainly between October and April (Ré *et al.*, 1990), at preferred temperatures of 14 and 15°C (Coombs *et al*. 2006). Sea surface temperatures above 17°C are clearly avoided (Stratoudakis *et al.*, 2007). Given that such temperatures occur mainly in the summer (see Picado *et al.*, 2013; Leitão *et al.*, 2014), they are not experienced by sardines during the early stages of development.

Sardine eggs and larvae were kept in recirculating systems and reared in 19 l cylindrical acrylic tanks placed inside 400 l water bath tanks in order to ensure thermally controlled conditions. Three replicates were established for each thermal treatment. Systems were filled with filtered (1 µm) and ultraviolet (UV)-irradiated natural seawater. Water quality was ensured by protein skimmers, bioballs and UV sterilizers, along with daily water changes. Concentrations of ammonia, nitrites and nitrates were monitored regularly and kept within recommended levels. Salinity was kept at 35.1 ± 0.4. Water temperatures (15.0 ± 0.4 and 17.1 ± 0.2°C) were monitored via a Profilux system connected to individual temperature probes. Temperature was automatically upregulated by heaters and downregulated using chillers.

Tanks were illuminated with a photoperiod of 14 h light–10 h dark. The wall of the tanks was covered with black plastic to reduce stress and increase prey visibility. Eggs were incubated at an initial density of 100 eggs l^−1^. Embryos hatched ∼48 h later. Larvae still had yolk reserves during the first days after hatching, with the mouth opening only at day 4. Larvae were fed *Gymnodinium* sp. (1500 individuals ml^−1^) on the day of hatching. Rotifers (*Brachionus plicatilis*) enriched with *Nannochloropsis* sp. (25 individuals ml^−1^) were introduced on day 3. Copepod (*Acartia tonsa*) nauplii enriched with *Rhodomonas baltica* were offered from day 4 at a density of 2 individuals ml^−1^, which was increased to 4 individuals ml^−1^ from day 20. Larvae were cultured in green water, by adding *Nannochloropsis* sp. twice a day.

### Hatching success, survival and growth

The hatching success was analysed in small rearing boxes placed inside the rearing tanks (three replicates per treatment). Ten eggs were randomly selected, placed inside each box and followed throughout the embryonic development. The hatching success was calculated as the percentage of eggs that hatched to normal larvae. At the end of the experiment, the survival of 30-day-old larvae was determined in each rearing tank.

Larval size was analysed at hatching (*n* = 60) and at the end of the 30 day acclimation period (*n* = 21). Larvae were randomly sampled, and their standard length was measured using a stereoscopic microscope. Growth rates were then determined as the difference between the mean length at hatching (*t*_0_) and the length of each larva at the end of the experiment (*t*_30_), divided by the number of days elapsed between *t*_0_ and *t*_30_.

### Metabolic rates

Oxygen consumption rates of sardine larvae were determined as an estimate of routine metabolic rates. Fish were last fed on the day before the respiration measurements, being deprived of food for ∼14 h to allow clearance of any food consumed and prevent the effects of feeding on routine metabolic rates. Thirty-day-old larvae were individually incubated in the respective thermal conditions (*n* = 6) in sealed respirometry chambers immersed in a temperature-controlled water bath in order to keep the temperature conditions stable over time. Each chamber was filled with 10 ml of filtered (0.2 µm) and UV-irradiated seawater in order to avoid bacterial respiration. Oxygen concentrations were recorded with Clark-type O_2_ electrodes connected to a Strathkelvin Instruments oxygen interface. Each respiratory run occurred after an acclimation period of 2 h (during which the high oxygen consumption rates caused by initial stress had decreased and stabilized) and lasted 4 h. Before and after each run, the experimental set-up was calibrated and checked for electrode drift and microbial oxygen consumption.

Thermal sensitivity (*Q*_10_) was determined using the following standard equation:

$$Q_{10}={\left(\frac{R_{2}}{R_{1}}\right)}^{\left(10/T_{2}-T_{1}\right)}$$

where *R*_1_ and *R*_2_ represent the oxygen consumption rate at temperatures *T*_1_ and *T*_2_, respectively.

### Thermal tolerance

Thirty-day-old larvae were incubated in glass containers with 100 ml of filtered (0.2 µm) and UV-irradiated seawater collected from the respective treatments. A total of three replicates (with five larvae each) were obtained per thermal treatment. These glass containers were suspended in a temperature-controlled water bath. The initial temperature was set to the acclimation temperature of the respective treatment and maintained for 30 min. Thereafter, temperature was increased at a rate of 1°C every 30 min. In each container, the seawater was aerated using air stones and the temperature was checked with thermocouple probes. Every 30 min, if no responsiveness was noticed, the larva was considered to be dead. The mean lethal temperature was determined for each container by averaging the temperatures at which each larva died.

### Behaviour

The behavioural analysis was performed based on the ethogram described in Table 1. Two behavioural categories were recorded: locomotion was evaluated using duration recording, whereas foraging was quantified using frequency recording. The behaviour of sardine larvae (12 larvae per treatment) was monitored through direct observation by an observer blinded to the treatment identity. Each larva was observed inside the rearing tank for 2 min, using the focal animal technique. Behavioural observations were made in the morning, 30 min after feeding.

**Table 1:** Ethogram of *Sardina pilchardus* larvae regarding locomotion and foraging behaviours

Category	Behaviour	Description
Locomotion	Inactive	The larva is motionless and stationary in the water column
	Swimming	The larva moves forward through the water column by stirring the caudal fin
Foraging	Orientation	The larva orients its body towards the prey and assumes an ‘S’-shaped positon, before trying an attack
	Attack	The larva moves forward towards the prey in an attempt to capture it

### Heat shock response and lipid peroxidation

#### Preparation of tissue extracts

At the end of the experimental period, 30-day-old larvae from each tank were pooled into different samples, placed immediately in liquid nitrogen and stored at −80°C. Samples were later homogenized in 100 µl of phosphate-buffered saline solution (0.14 M NaCl, 2.7 mM KCl, 8.1 mM Na_2_HPO_4_ and 1.47 mM KH_2_PO_4_, pH 7.4), by using a glass tissue grinder with a teflon pestle. All homogenates were centrifuged for 20 min at 14 000 ***g*** at 4°C. The supernatant fraction was then used to assess the heat shock response and lipid peroxidation in sardine larvae exposed to the different thermal scenarios. A total of six replicates were obtained per treatment.

#### Quantification of heat shock response

The heat shock response of sardine larvae was estimated based on the production of the heat shock proteins HSP70/HSC70 (both constitutive and inducible forms), which was determined by enzyme-linked immunosorbent assay, based on Njemini *et al.* (2005). In brief, a total of 10 µl of the supernatant fraction of the homogenate was diluted in 990 µl of phosphate-buffered saline. Then, 50 µl of the diluted sample was added in triplicates to 96-well microplates and incubated overnight at 4°C. Microplates were washed on the next day in phosphate-buffered saline (containing 0.05% Tween-20). A total of 100 µl of blocking solution (1% bovine serum albumin) was added to each well, and the microplates were then incubated for 2 h at room temperature and in darkness. After that, 50 µl of a solution of 5 µg ml^−1^ of primary antibody anti-HSP70/HSC70 (detecting both 72 and 73 kDa proteins, which correspond to the molecular mass of inducible HSP70 and constitutive HSC70, respectively) was added to each well. Then, microplates were incubated overnight at 4°C. The non-linked antibodies were removed on the next day by washing the microplates. The alkaline phosphatase-conjugated anti-mouse IgG (Fab specific) was then used as a secondary antibody, by adding 50 µl of a solution at 1 µg ml^−1^ to each well and incubating the microplates for 90 min at 37°C. After another wash, 100 µl of substrate *p*-nitrophenyl phosphate tablets was added to each well and incubated for 10–30 min at room temperature. Subsequently, 50 µl of stop solution (3 M NaOH) was added to each well, and the absorbance was read at 405 nm in a 96-well microplate reader. The amount of HSP70/HSC70 in the samples was then calculated from a standard curve of absorbance based on serial dilutions (from 30 to 1000 ng ml^−1^) of purified HSP70/HSC70 active protein. The results were expressed in relation to the protein content of the samples, which was determined according to the Bradford method (see Bradford, 1976).

#### Quantification of lipid peroxidation

The lipid peroxidation in sardine larvae was evaluated based on the quantification of malondialdehyde (MDA), a specific end-product of the oxidative degradation process of lipids. The TBARS (thiobarbituric acid reactive substances) assay (adapted from Uchiyama and Mihara, 1978) was used to quantify MDA. The thiobarbituric acid reacts with the MDA to yield a fluorescent product, which can then be detected spectrophotometrically. A total of 10 µl of the supernatant fraction of the homogenate was treated with 12.5 µl of 8.1% sodium dodecyl sulphate, 93.5 µl of 20% trichloroacetic acid (pH 3.5) and 93.5 µl of 1% thiobarbituric acid. A volume of 50.5 µl of Milli-Q ultrapure water was added to this mixture, which was then mixed in a vortex for 30 sec and incubated in boiling water for 10 min. The mixture was placed on ice for 3 min to lower the temperature. Afterwards, 62.5 µl of Milli-Q ultrapure water and 312.5 µl of n-butanol pyridine (15:1 v/v) were added, and the mixture was placed in a vortex and centrifuged at 2000 *g* for 5 min. A total of 150 µl of the supernatant fraction was added in duplicates to 96-well microplates, and the absorbance was read at 532 nm. The MDA concentrations in the samples were calculated based on a calibration curve (from 0 to 0.3 µM) using MDA *bis*(dimethyl acetal) standards. The results were expressed in relation to the protein content of the samples, which was determined according to the Bradford method (see Bradford, 1976).

### Statistical analysis

All data were analysed using generalized linear mixed-effects models (see e.g. Zuur *et al*., 2009). The distributional family used was binomial (logit link function) for proportions (i.e. hatching and survival), Poisson (log link function) for counts (i.e. attack frequency), Gaussian (identity link function) for quantities with linear response (i.e. length, growth, oxygen consumption rate, lethal temperature, swimming duration and HSP) and Gamma (inverse link function) for positive quantities with a severe positively skewed distribution (i.e. MDA). The mixed models included the temperature as a fixed effect and the rearing tank as a random effect to account for possible dependency within tanks. Following the recommendation from Barr *et al.* (2013), the random effects were kept in the models irrespective of the amount of variation they explained. Model residuals were checked for departures from the assumed distributions, and no significant deviations were found. Effect sizes (ES) were estimated using odds ratios for binomial models and the ratio between the fixed effect coefficient and the residual standard error for the remaining models.

All statistical analyses were implemented in R (R Core Team, 2015), using the lme4 (Bates *et al.*, 2015) and nlme (Pinheiro *et al.*, 2015) packages. Results were considered statistically significant at a significance level of 0.05.

## Results

### Hatching success, survival and growth

**Figure 1:**
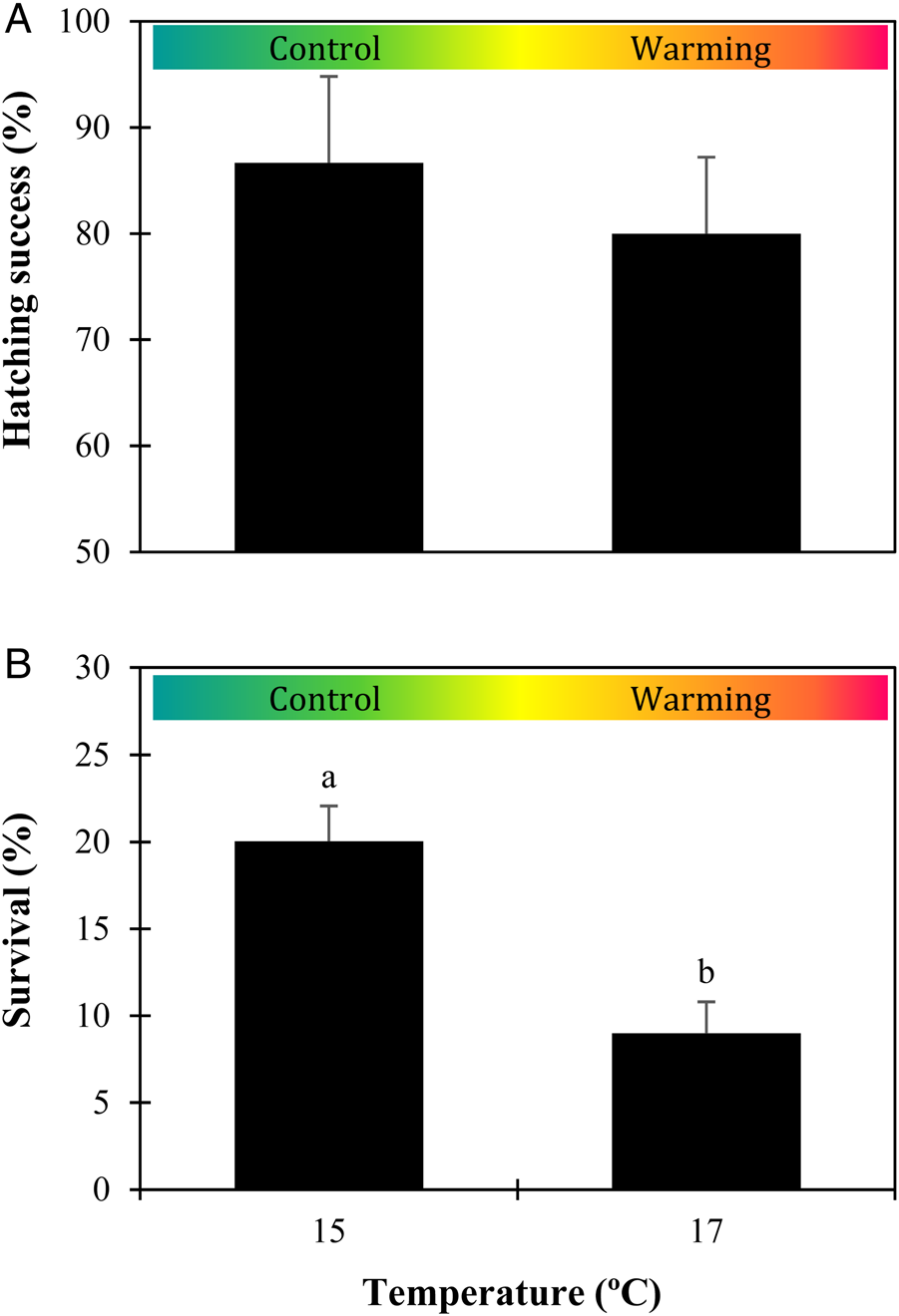
Impact of warming on the hatching success and survival of *Sardina pilchardus* larvae. Hatching rate of embryos (**A**) and survival rate of 30-day-old larvae (**B**) in different thermal scenarios. Values represent means + SD (*n* = 3). Different letters represent significant differences between thermal treatments (*P* < 0.05).

The hatching success of sardine embryos (Fig. 1A) was not significantly affected by warming (*P* = 0.209), varying from 86.7 ± 8.1% at the present-day temperature to 80.0 ± 7.2% under the future warming scenario. Accordingly, the odds ratio was 0.58 (95% confidence interval: 0.25, 1.35), revealing a small effect size. The survival of 30-day-old larvae (Fig. 1B) decreased significantly with warming (*P* < 0.001), from 20.0 ± 2.0 to 9.0 ± 1.8%. In this case, the odds of surviving in the warm conditions were 0.39 (95% confidence interval: 0.31, 0.49) times the odds at the control temperature, i.e. the odds of surviving were 2.6 times higher at the control temperature.

**Figure 2:**
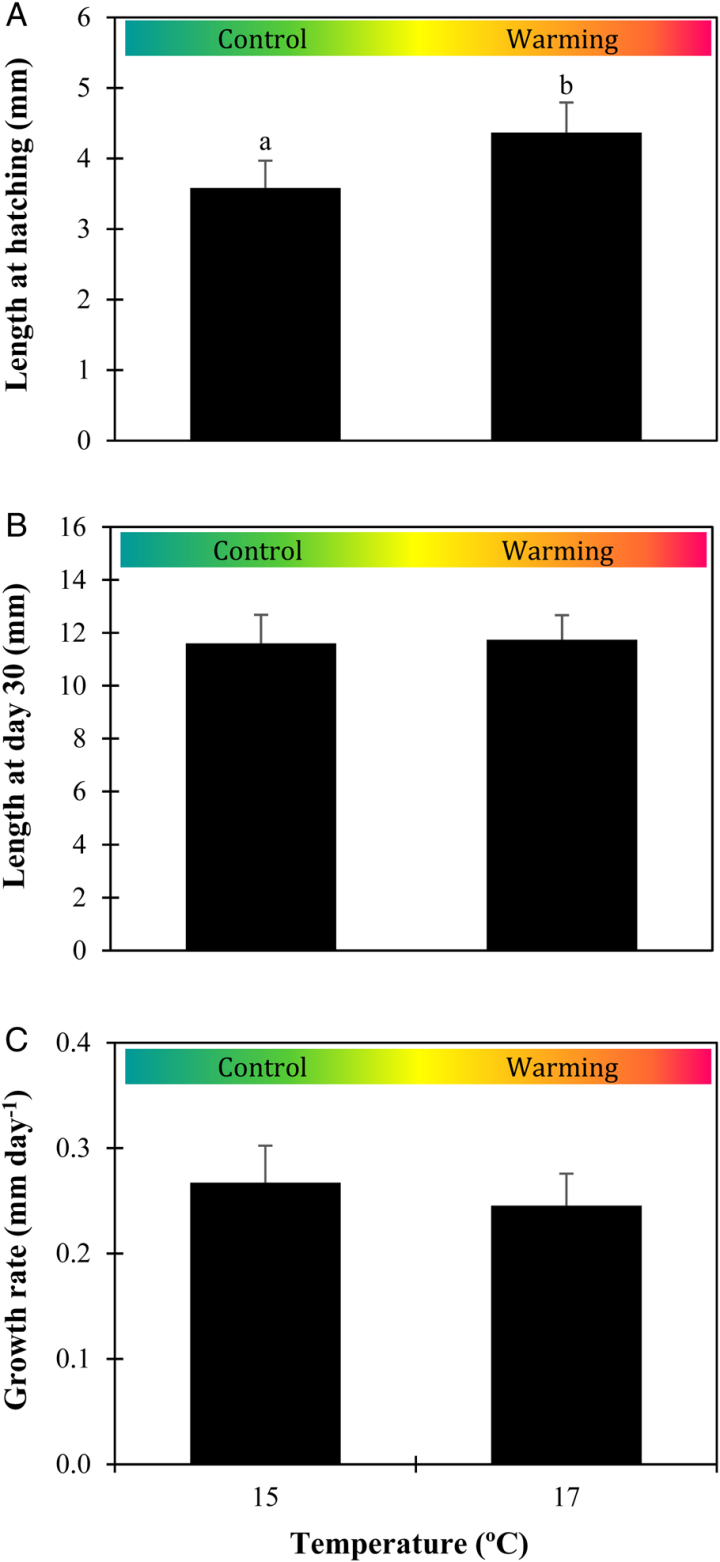
Impact of warming on the size of *Sardina pilchardus* larvae. Standard length of newly hatched larvae (**A**), standard length of 30-day-old larvae (**B**) and growth rate of 30-day-old larvae (**C**) in different thermal scenarios. Values represent means + SD (*n* = 60 for newly hatched larvae; *n* = 21 for 30-day-old larvae). Different letters represent significant differences between thermal treatments (*P* < 0.05).

Higher temperature had a significant and positive effect on the length of newly hatched larvae (*P* = 0.002, ES = 2.1), but not on the length of 30-day-old larvae (*P* = 0.695, ES = 0.1). At hatching, larval length was 3.6 ± 0.4 and 4.4 ± 0.4 mm at control temperature and warming, respectively (Fig. 2A). At the end of the experiment, the length of 30-day-old larvae varied between 11.6 ± 1.1 and 11.7 ± 0.9 mm at the lower and higher temperature, respectively (Fig. 2B). No significant effect of temperature was found on larval growth during the first 30 days of life (*P* = 0.112, ES = 0.7), varying between 0.25 ± 0.03 and 0.27 ± 0.04 mm day^−1^ in the warm and control conditions, respectively (Fig. 2C).

### Metabolic rates and thermal tolerance

**Figure 3:**
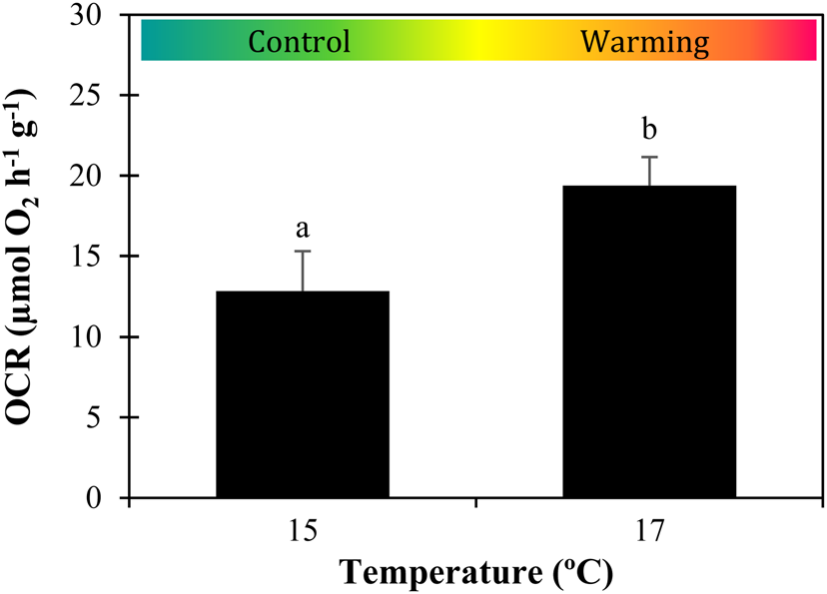
Impact of warming on the metabolism of *Sardina pilchardus* larvae. Oxygen consumption rate (OCR) of 30-day-old larvae in different thermal scenarios. Values represent means + SD (*n* = 6). Different letters represent significant differences between thermal treatments (*P* < 0.05).

Warming elicited a significant increase in the metabolism of sardine larvae (*P* = 0.006, ES = 3.3), from 12.8 ± 2.5 to 19.4 ± 1.8 µmol O_2_ h^−1^ g^−1^ (Fig. 3). This metabolic increment resulted in a *Q*_10_ value of 7.9.

**Figure 4:**
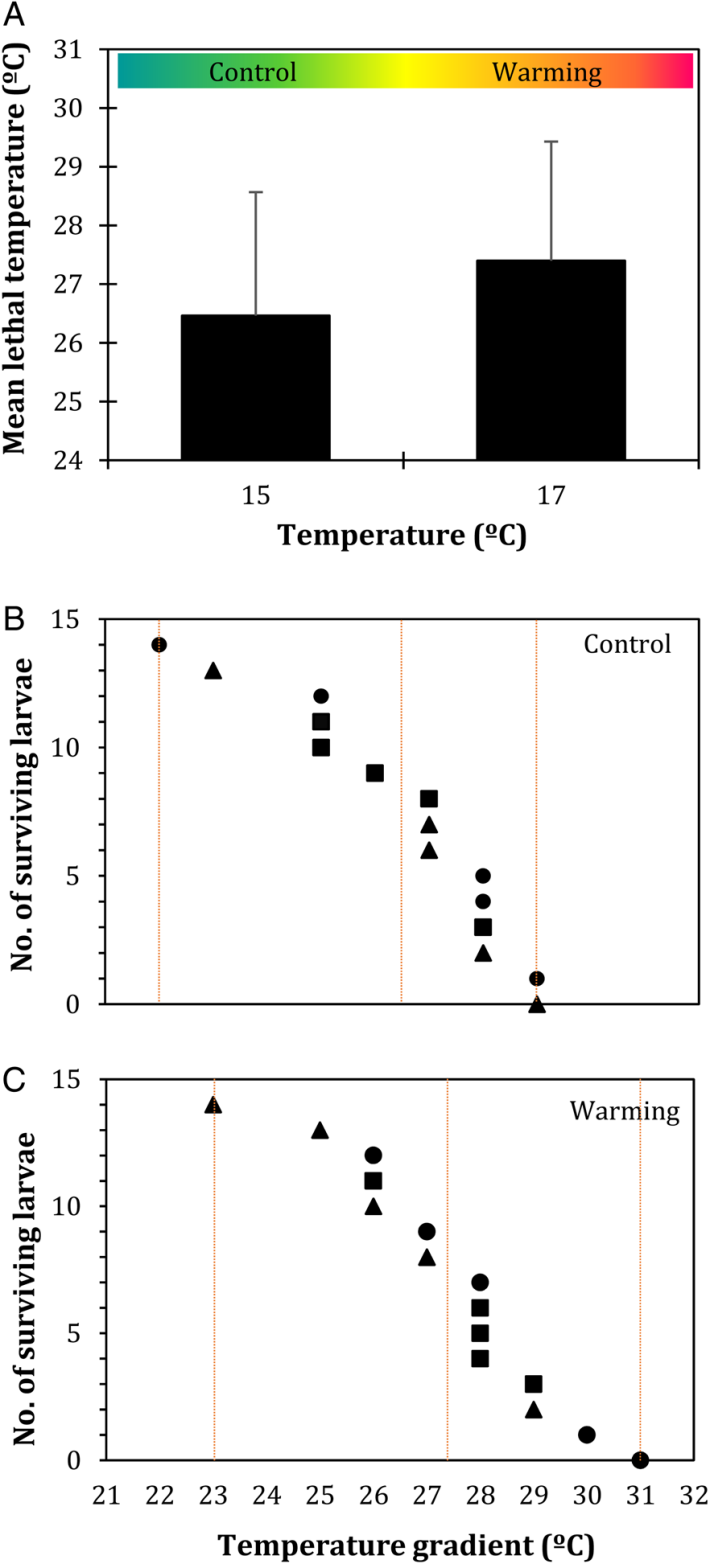
Impact of warming on the thermal tolerance of *Sardina pilchardus* larvae. (**A**) Mean lethal temperature of 30-day-old larvae in different thermal scenarios. Values represent means + SD (*n* = 3). (**B** and **C**) Number of surviving larvae from the control temperature and warming conditions, respectively, after exposure to an increasing temperature gradient. Different symbols represent different replicates. Vertical lines indicate the temperature at which larvae start dying, the mean lethal temperature and the temperature at which larvae stop dying. No significant differences were found between thermal treatments (*P* > 0.05).

The thermal tolerance of sardine larvae (Fig. 4) was not significantly affected by warming (*P* = 0.283, ES = 0.5). However, it is worth mentioning that the mean lethal temperature was ∼1°C higher in the warm conditions (27.4 ± 2.1°C) than at the control temperature (26.5 ± 2.0°C). Moreover, larvae exposed to warming started to die later (at 23 instead of 22°C) and stopped dying at higher temperatures (at 31 instead of 29°C).

### Behaviour

**Figure 5:**
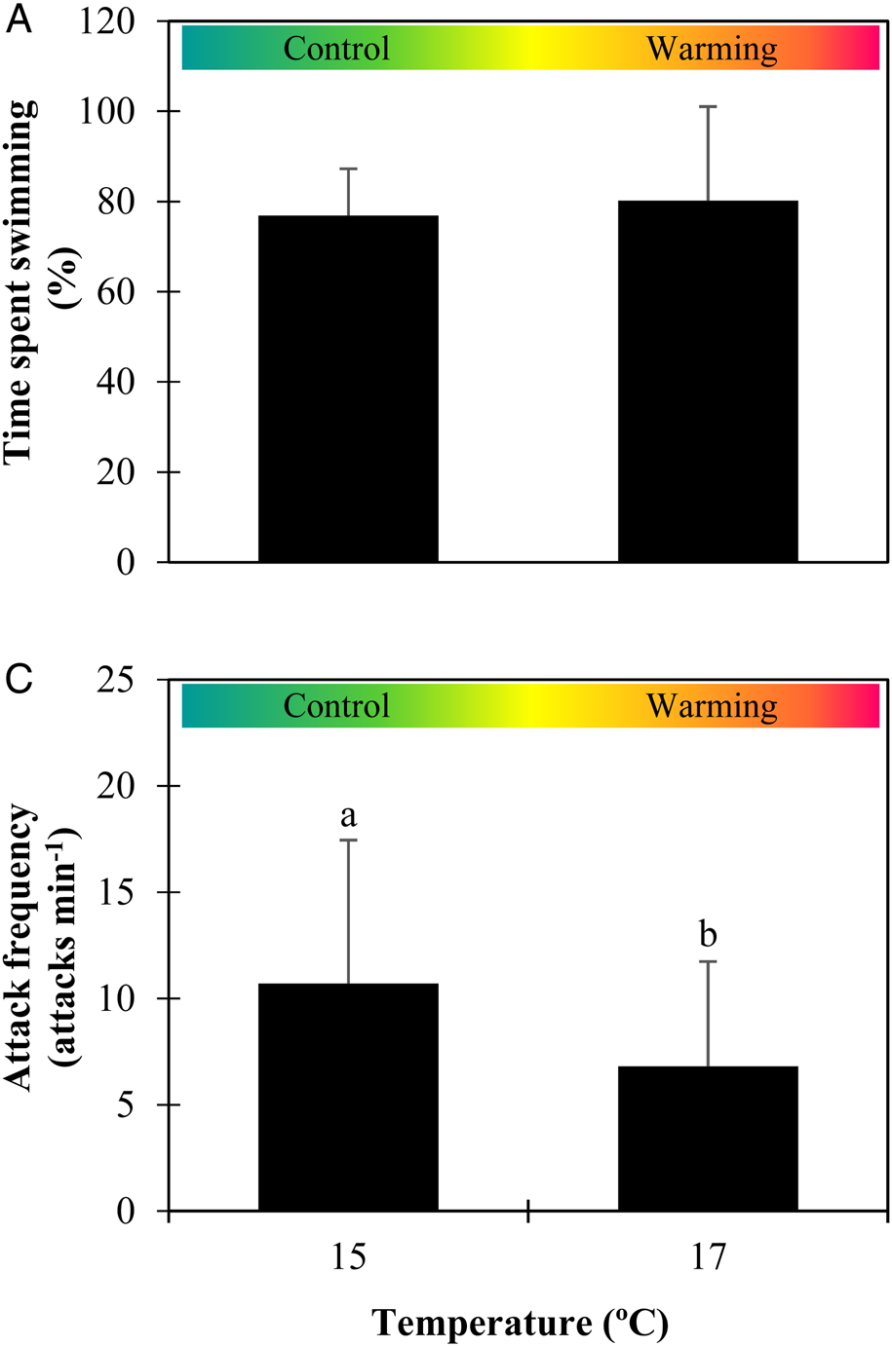
Impact of warming on the locomotion and foraging behaviours of *Sardina pilchardus* larvae. Time spent swimming (**A**) and frequency of prey attacks (**B**) of 30-day-old larvae in different thermal scenarios. Values represent means + SD (*n* = 12). Different letters represent significant differences between thermal treatments (*P* < 0.05).

No significant differences (*P* = 0.257, ES = 0.6) were found in the locomotion patterns (Fig. 5A). Sardine larvae spent 76.9 ± 10.4 and 80.2 ± 20.9% of the time swimming at the lower and higher temperature, respectively. With regard to the foraging behaviour, 100.0 and 98.6% of the orientations towards the prey resulted in attacks at the lower and higher temperature, respectively. The frequency of prey attacks (Fig. 5B) decreased significantly with warming (*P* = 0.007, ES = 4.0), from 10.8 ± 6.8 to 6.8 ± 4.9 attacks min^−1^.

### Heat shock response and lipid peroxidation

**Figure 6:**
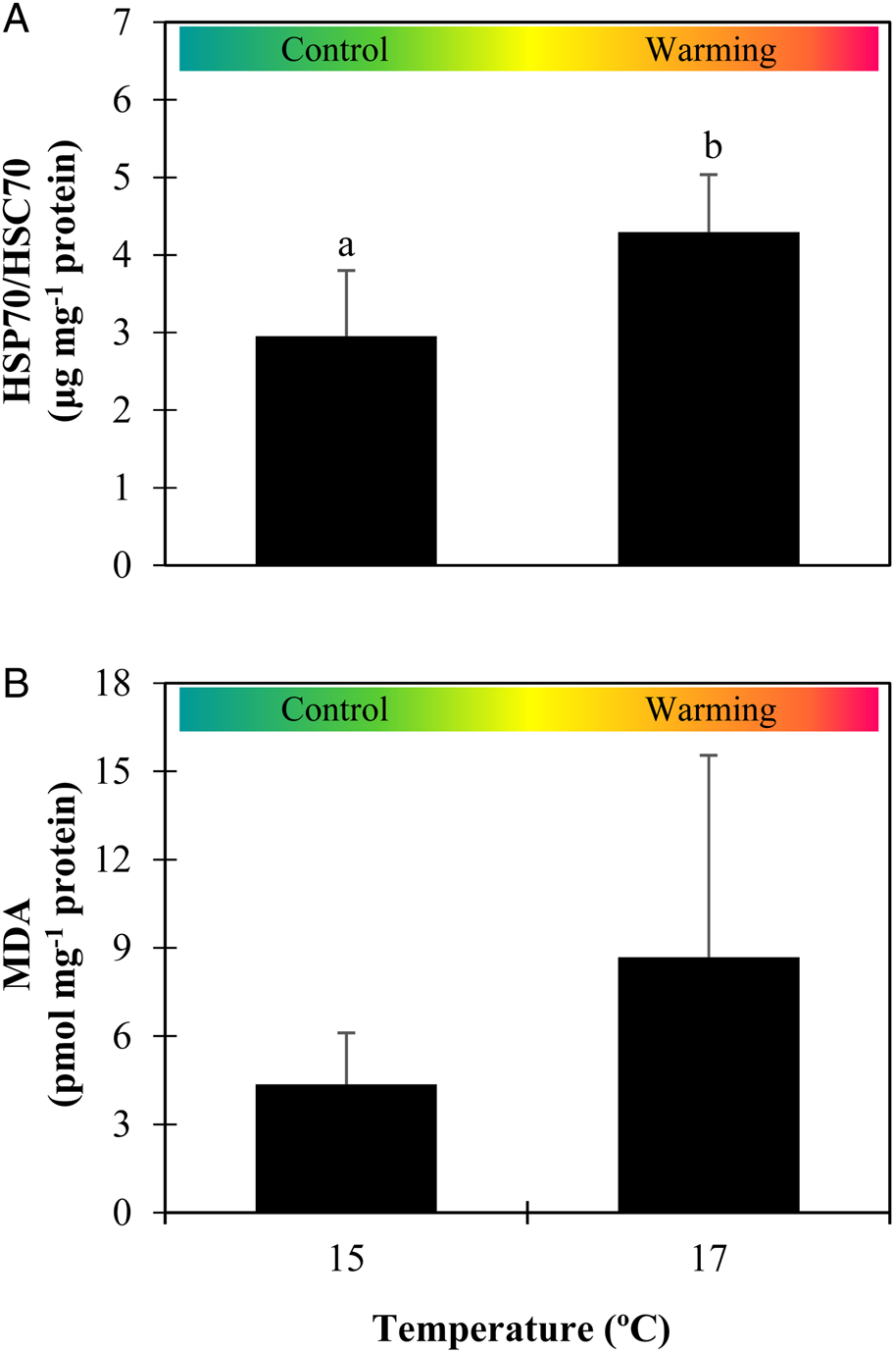
Impact of warming on the heat shock response and lipid peroxidation of *Sardina pilchardus* larvae. Heat shock protein (HSP70/HSC70) levels (**A**) and malondialdehyde (MDA) concentrations (**B**) of 30-day-old larvae in different thermal scenarios. Values represent means + SD (*n* = 6). Different letters represent significant differences between thermal treatments (*P* < 0.05).

The warming scenario had a significant impact on the heat shock response of sardine larvae (Fig. 6A). The levels of HSP70/HSC70 increased significantly at the higher temperature (*P* = 0.043, ES = 1.9), from 3.0 ± 0.9 to 4.3 ± 0.7 µg (mg protein)^−1^.

Lipid peroxidation (Fig. 6B) was not significantly affected by warming (*P* = 0.333, ES = 1.3). Nevertheless, mean MDA levels nearly doubled, from 4.4 ± 1.7 pmol (mg protein)^−1^ at lower temperatures to 8.7 ± 6.7 pmol (mg protein)^−1^ at higher temperatures.

## Discussion

The distribution and abundance of *S. pilchardus* is well known to fluctuate with seawater temperature (e.g. Coombs *et al.*, 2006; Planque *et al.*, 2007; Bellido *et al.*, 2008; Alheit *et al.*, 2012; Santos *et al.*, 2013; Montero-Serra *et al.*, 2015), but it remains unclear how higher temperatures affect the biology of this species. This study shows that warming may have a negative effect on several biological aspects of the early ontogeny of the European sardine.

During the short embryonic development of this species (∼2 days), warming did not have a significant effect on the hatching success. However, with continuous exposure to warming, the impact of higher temperatures became more evident. The survival of 30-day-old larvae was reduced by half in the warmer conditions. Moreover, warming was responsible for increasing the size of newly hatched larvae as expected, but did not affect the size and growth of 30-day-old larvae. Given that temperature is well known to accelerate development, the lack of higher growth rates at warmer temperatures may suggest a certain degree of physiological stress. In stressful conditions, part of the energy budget has to be allocated away from non-essential processes, such as growth and reproduction, towards primary processes, including those involved in stress management (Wieser, 1994).

Sardine larvae exhibited signs indicative of thermal stress when exposed to warming, namely a pronounced increase of larval metabolism (*Q*_10_ value of 7.9, far beyond the normal range of 2–3 for most ectotherms; Hochachka and Somero, 2002) and increased heat shock response. Exposure to environmental stress may be compensated by physiological protective mechanisms, such as the heat shock response, which help to reduce the negative effects of stress on larval fitness. The increased metabolic demands faced by the early stages under warming are expected to increase the formation of reactive oxygen species (ROS), which may injure cellular mechanisms by lipid peroxidation, one of the most frequent cellular injury mechanisms, where ROS react with membrane-associated lipids (Lesser, 2011). Heat shock proteins are responsible for repairing, refolding and eliminating damaged or denatured proteins (Sung and MacRae, 2011) and are also among the molecules that can induce protection against ROS (Polla *et al.*, 1996). In the present study, the heat shock response of sardine larvae was enhanced by 45% in the warmer conditions. This enhanced response may have helped, among other possible things, to minimize the effects of ROS and the respective levels of lipid peroxidation at higher temperatures. The build-up of MDA was not significantly affected by warming, even though the mean levels had doubled.

The impact of warming and thermal stress was not, however, noted in the locomotion patterns of sardine larvae. No signs of increased activity or lethargy were detected at the high temperature. However, with regard to foraging behaviour, the frequency of prey attacks decreased by 36% with warming, which may have important consequences for fish condition. In warmer conditions, feeding rates are expected to follow the increase in metabolic rates, in order to fuel the greater metabolic costs at higher temperatures (Rosa and Seibel, 2010). Lower feeding rates, resultant from a lower number of prey attacks, will probably be insufficient to satisfy the greater energetic demands at warmer temperatures.

Overall, warming had a negative effect on the early stages of *S. pilchardus*, namely on their survival, metabolism, feeding behaviour and stress response. In nature, reduced chances of larval survival are expected to affect fish recruitment and further impact the lower and higher levels of the marine trophic chain. Given the great complexity of marine ecosystems, other factors should, however, be considered when evaluating the impact of future climate change. For instance, the present study did not consider the impact that ocean warming may have on the primary productivity of oceans and, consequently, on the abundance of planktonic organisms and prey availability. Moreover, ocean warming is predicted to occur simultaneously with other environmental stresses, such as ocean acidification and the expansion of hypoxic zones (Pörtner *et al.*, 2014). These threats have been shown to have synergistic effects on marine organisms (e.g. Rosa and Seibel, 2008; Munday *et al.*, 2009; Kroeker *et al.*, 2013; Pimentel *et al.*, 2014; Faleiro *et al.*, 2015), which may magnify the challenge for marine life. Nevertheless, it is important to keep in mind that, despite the potentially negative effects of climate change, there will be some scope for acclimation and adaptation (Munday, 2014). In the present study, even though the thermal tolerance of sardine larvae was not significantly affected by warming, it is worth mentioning that larvae exposed to warmer temperatures started and stopped dying at higher temperatures, presenting a mean lethal temperature ∼1°C higher. Until the end of the century, marine organisms will have more time and evolutionary opportunities to adapt to future changes in the ocean’s climate.

## Funding

This study was financially supported by the Portuguese Foundation for Science and Technology. The experiments were carried out as part of the project EARLY CHANGE (PTDC/AAG-GLO/3342/2012, with R.R. as Principal Investigator). The obtainment of sardine eggs and the maintenance of the trophic chain in captivity were supported by the project VITAL (PTDC/MAR/111304/2009). Researchers were funded by a post-doctoral grant to F.F. (SFRH/BPD/79038/2011) and doctoral grants to M.P. (SFRH/BD/81928/2011) and A.R.L. (SFRH/BD/97070/2013).
